# Usefulness of intra-operative neuromuscular blockade monitoring and reversal agents for postoperative residual neuromuscular blockade: a retrospective observational study

**DOI:** 10.1186/s12871-019-0817-4

**Published:** 2019-08-07

**Authors:** Gonzalo Domenech, Matías A. Kampel, María E. García Guzzo, Delfina Sánchez Novas, Sergio A. Terrasa, Gustavo Garcia Fornari

**Affiliations:** 1 Department of Anesthesiology, Italiano de Buenos Aires Hospital, Presidente Teniente General Juan Domingo Perón 4190, Postal Code, 1199 Buenos Aires, Argentina; 2 Department of Research, Italiano de Buenos Aires Hospital, Buenos Aires, Argentina

**Keywords:** Neuromuscular blockade, Residual neuromuscular blockade, Neuromuscular blockade monitoring

## Abstract

**Background:**

Complete avoidance of residual neuromuscular blockade (RNMB) during the postoperative period has not yet been achieved in current anesthesia practice. Evidently, compliance with NMB monitoring is persistently low, and the risk of RNMB during the perioperative period remains underestimated. To our knowledge, no publications have reported the incidence of RNMB in a university hospital where access to quantitative NMB monitoring and sugammadex is unlimited and where NMB management is not protocolised. The primary aim of this study was to estimate the incidence of RNMB in patients managed with or without sugammadex or neostigmine as antagonists and quantitative NMB monitoring in the operating room. The secondary aim was to explore the associations between RNMB and potentially related variables.

**Methods:**

This retrospective observational cohort study was conducted at a tertiary referral university hospital in Buenos Aires, Argentina. Records created between June 2015 and December 2015 were reviewed. In total, 240 consecutive patients who had undergone elective surgical procedures requiring NMB were included. All patients were monitored via acceleromyography at the adductor pollicis muscle within 5 min of arrival in the postanaesthesia care unit (PACU). Scheduled recovery in the intensive care unit was the only exclusion criterion.

**Results:**

RNMB was present in 1.6% patients who received intra-operative quantitative NMB monitoring and 32% patients whose NMB was not monitored (*P* <  0.01). Multivariable analysis revealed that the use of intra-operative quantitative NMB monitoring and sugammadex were associated with a lower incidence of RNMB, with calculated odds ratios of 0.04 (95% confidence interval [CI]: 0.005 to 0.401) and 0.18 (95% CI: 0.046 to 0.727), respectively.

**Conclusions:**

The results of the present study suggest that intra-operative quantitative NMB monitoring and use of sugammadex are associated with a decreased incidence of RNMB in the PACU, reinforcing the contention that the optimal strategy for RNMB avoidance is the use of quantitative NMB monitoring and eventual use of reversal agents, if needed, prior to emergence from anaesthesia.

## Background

Complete avoidance of residual neuromuscular blockade (RNMB) during the postoperative period has not yet been achieved*.* The clinical implications of RNMB are not clearly defined, but several studies suggest that it can prolong postoperative recovery, adversely affect respiratory function, impair airway-protective reflexes, and induce unpleasant symptoms associated with muscle weakness [1]. RNMB is currently defined as the presence of a train-of-four (TOF) ratio (TOFR; ratio of the fourth to the first twitch height) of < 0.9 (90%) [2]. The reported incidence varies from 20 to 50% in the postanaesthesia care unit (PACU), depending on the TOFR threshold (0.7 or 0.9) used to define RNMB in each study, type of neuromuscular blocking drugs (NMBDs) used, and the use of objective or intra-operative quantitative monitoring of neuromuscular function [3].

Despite the high incidence of RNMB reported in modern PACUs, reported as high as 45% [4], awareness of its clinical implications remains limited. In an online survey, 15% of anaesthesiologists in the United States and Europe reported that they had never observed an episode of RNMB in their patients [5]. Recently, a consensus statement on the perioperative use of NMB monitoring strongly recommended the use of quantitative NMB monitoring whenever a nondepolarising NMBD is administered [6]. Also, several reports suggest that clinical evaluation and qualitative neuromuscular monitoring approaches are not sensitive enough to detect the presence of RNMB [7–9].

In 2 previous studies, the selective agent γ-cyclodextrin sugammadex was proven to eliminate RNMB in the PACU among patients receiving intra-operative quantitative NMB monitoring and to accelerate reversal times when compared with neostigmine [10, 11]. In another study, however, sugammadex reportedly failed to prevent postoperative TOFR values of <  0.9 without the concomitant use of intra-operative NMB monitoring [12].

To date, no publications have reported the incidence of RNMB in a hospital where access to quantitative NMB monitoring and sugammadex is unlimited and where NMB management is not protocolised. Regarding this matter, our hypothesis was that our PACU would have an incidence of RNMB as high as 45%, as reported by Debaene B, et al. [13] and that both intra-operative quantitative NMB monitoring and the use of reversal agents would be associated with a lower incidence of RNMB.

### Objectives

#### Primary aim

To estimate the incidence of RNMB in patients managed with or without sugammadex and neostigmine as antagonists, and quantitative NMB monitoring in the operating room.

#### Secondary aim

To explore the associations between RNMB and potentially related variables such as the use of NMB monitoring, duration of surgery, type of NMBD, type of reversal agent, and time between the last NMBD administration and TOFR in the PACU.

## Methods

Ethical approval for this retrospective cohort study was provided by the Ethical Committee of the Hospital Italiano de Buenos Aires in Buenos Aires, Argentina (Chairperson: Dr. Augusto Pérez, Ethical Committee N° 2832) on June 10, 2016. This study was conducted at the Italiano de Buenos Aires Hospital, a tertiary referral university hospital in Buenos Aires, Argentina. A medical record review of 240 consecutive patients who had undergone elective surgical procedures requiring NMB was performed. Scheduled recovery in the intensive care unit was the only exclusion criterion. Acceleromyography (AMG) in the PACU was routine clinical practice during 2015 as part of a clinical audit to improve patient care. Cases between June 2015 and December 2015 were reviewed. The jurisdictional Institutional Review Board that approved the study waived the need for informed consent from the patients due to the retrospective nature of the study.

The patient variables recorded included sex, age, the American Society of Anesthesiologists (ASA) physical status classification score, weight, height, and body mass index. The surgery-related data assessment included duration of surgery, NMBD used (compound, total dose, and time from last dose to TOFR in the PACU), antagonist for NMBD (sugammadex or neostigmine), and the use of quantitative neuromuscular function monitoring. All data were obtained via electronic medical records.

Two different types of NMB monitoring were used due to limited availability of monitors. Intra-operative TOFR measurements were determined via a Philips IntelliVue NMT Module 865,383 (Phillips Healthcare, Amsterdam, the Netherlands), attached to multiparameter monitors in every operating room as it is not portable. A different monitor, TOF-Watch® SX (Organon Ireland Ltd., a division of MSD, Dublin, Ireland), was available as a portable device for PACU measurements. Although both monitors use the same measuring principle (AMG), there is no published evidence on the similarity of measurements between them. No qualitative monitors were used during the study. The site of monitoring as well as the results of quantitative monitoring (depth of block and TOFR prior to tracheal extubation) were not manually recorded nor automatically transferred to the electronic anaesthesia record. The retrospective nature of the study precluded possible methods for obtaining this information. Results from TOFRs before tracheal extubation were verbally communicated to the physician performing TOF monitoring in the PACU in cases in which NMB monitoring or reversal agents had been used. Results from TOFRs in the PACU were added manually in the postanaesthetic subsection of the electronic anaesthesia record.

Within 5 min of arriving in the PACU, all patients were monitored via AMG of the adductor pollicis muscle (TOF-Watch® SX). TOFR measurements were performed by anaesthesia trainees who had previous training in NMB monitoring. Surface electrodes were placed 3 cm apart over the ulnar nerve as routine practice. The skin was prepared using alcohol cleansing pads to decrease resistance to stimulation. Uncalibrated TOF stimulation was applied and free movement of the thumb was ensured in the monitored extremity. Electrode placement for TOF monitoring was standardised, and it was not manually recorded. Nonetheless, it is highly likely that NMB monitors were applied to the thumb opposite existing peripheral venous or arterial catheters in both the operating room and the PACU.

The stimulation current was set at 50 mA (4 pulses of 0.2 ms duration at a frequency of 2 Hz) and 3 consecutive stimuli were applied to each patient, separated by 15 s. Baseline settings of the TOF Watch SX monitor include a stimulating current of 50 mA*.* The mean of the 3 resulting TOFRs was used for decision making. An NMBD antagonist (sugammadex or neostigmine) was administered if the TOFR was < 0.9.

The primary outcome was the presence of RNMB, defined as a TOFR of < 0.9. The secondary outcomes were the associations between RNMB and potentially related variables as per a multivariable regression model.

### Statistical analysis

We assumed a 45% incidence of RNMB [13] and calculated a required sample size of 222 patient records. This sample size allowed an incidence estimation with a 95% confidence interval (CI) margin of error that did not exceed 7%. Eventually, 240 electronic medical records were analysed after considering a missing data rate of 10%.

Descriptive analyses were performed using the mean ± standard deviation for continuous variables and numbers and percentages for categorical variables. Qualitative variables derived from each group were compared using the chi-square test or Fisher’s exact test in cases involving low expected counts. The Student’s *t*-test was used to analyse normally distributed quantitative data, while the nonparametric Wilcoxon rank-sum test was used to analyse non-normally distributed quantitative data.

In the first step, we evaluated the association between the different potentially predictive variables and the outcome (RNMB) through a bivariate analysis. Since we had a total of 58 outcomes, we predicted that we could build a multivariable model with a maximum of 5 potential predictors as recommended by Norman et al. [14]

The multivariable model was built using the 4 variables (use of monitoring, type of reversal agent, duration of surgery, and the time between the last administration of NMBD and the presence of a TOFR in the PACU) that had shown a statistically significant association (*P* <  0.1) in the bivariate analysis. In addition, we forced the entry of the NMBD type as a variable for clinical reasons.

Once the model was built, we tested whether the elimination of those variables with no statistical significance (*P* > 0.05) modified the odds ratio (OR) of the other variables in the model in a substantial way (more than 10%). For this reason, the duration of surgery was retained as a variable in the model.

The final model was established using the following variables: use of NMB monitoring, type of reversal agent used, duration of surgery, time between the last administration of NMBD and TOFR in the PACU, and type of NMBD used.

## Results

A total of 240 patients were included in this retrospective observational study. The overall incidence of RNMB was 24% (58/240). One of 63 patients (1.6%) who received intra-operative quantitative NMB monitoring and 57 of 177 (32%) patients who were not monitored exhibited RNMB (*P* <  0.01; Table 1). The mean TOFR in patients who exhibited RNMB (TOFR < 0.9) was 0.68 ± 0.20, while that in patients without RNMB was 0.94 ± 0.03. The incidence of a TOFR of < 0.7 was 10%.

**Table 1:** Patient demographics and anaesthetic variables

	Nonmonitored group (*n* = 177)	Monitored group (*n* = 63)	*P* value
Age (years)	53.3 ± 17.82	57 ± 16.66	0.15┴
Sex (male/female)	60/117	29/34	0.087*
Body mass index	26.4 ± 5.16	28.1 ± 5.97	0.029┴
ASA physical status (I/II/III/IV)	17/123/37/0	6/36/19/2	0.05^#^
Duration of surgery (min)	142.3 ± 67.20	154.8 ± 65.69	0.204┴
NMBD used			
Vecuronium	7	1	0.804^#^
Atracurium	42	14	
Rocuronium	128	48	
Interval between last NMBD administration and TOFR measurement (min)	134.43 ± 61.66	105.33 ± 61.08	0.014┴
NMB antagonist, n/total (%)	24/177 (13.5)	51/63 (81)	< 0.01*
Neostigmine	5/24	9/63	< 0.01*
Sugammadex	19/24	42/63	
Dose of NMB antagonist			
Neostigmine (μg kg^−1^)	29.51 ± 9.83	29.64 ± 1.06	0.98┴
Sugammadex (mg kg^− 1^)	3.22 ± 1.23	3.90 ± 2.66	0.29┴
RNMB, n/total (%)	57/177 (32%)	1/63 (1.6%)	< 0.001

Data are expressed as the mean ± standard deviation or numbers

*ASA* American Society of Anesthesiologists, *NMB* Neuromuscular blockade, *NMBD* Neuromuscular blocking drug, *RNMB* Residual neuromuscular blockade, *TOFR* Train-of-four ratio

┴Student’s t-test

*Chi-square test

#Fisher’s exact test

Demographic patient characteristics are presented in Table 1. Intra-operative quantitative NMB monitoring was used in 63 of 240 patients (26%). There were no significant differences pertaining to age, sex, body mass index, ASA physical status, or type of NMBD used in both groups. The mean duration of surgery was 142.3 ± 67.20 min in the nonmonitored group and 154.8 ± 65.69 min in the monitored group (*P* = 0.204).

Rocuronium, vecuronium, and atracurium were administered to 73, 23, and 3% patients with mean doses of 54.15 ± 29.06, 8.23 ± 6.52, and 41.87 ± 16.89 mg, respectively (Table 2). All patients who received atracurium as an NMBD were reversed with neostigmine and all patients who received sugammadex were under NMB after rocuronium or vecuronium administration.

**Table 2:** Nondepolarising neuromuscular blockade management

	Agent	*n*/total (%)	Total dose (mg)	Total dose (mg kg^− 1^)
Nondepolarising neuromuscular blockade	Rocuronium	176/240 (73%)	54.15 ± 29.06	0.61 ± 0.18
	Vecuronium	56/240 (23.3%)	8.23 ± 6.52	0.08 ± 0.025
	Atracurium	8/240 (3.3%)	41.87 ± 16.89	0.42 ± 0.067

*n* Number of patients

Data are presented as the mean ± standard deviation or numbers

Among the 63 monitored patients, 9 received neostigmine and 42 received sugammadex prior to tracheal extubation (51/63, 81%). Five and 19 of the 177 nonmonitored patients received neostigmine and sugammadex (24/177, 13.5%), respectively. Postoperative RNMB was present in 2 of the 5 patients (40%) who received neostigmine without quantitative NMB monitoring and none of the patients who received neostigmine with quantitative NMB monitoring (*P* = 0.11). Sugammadex failed to reverse the blockade in 3 of 19 patients (16%) in the nonmonitored group and none of the patients in the monitored group (*P* = 0.028; Table 3).

**Table 3:** Neuromuscular blockade (NMB) monitoring and the use of NMB antagonists

	Intra-operative NMB monitoring (*n* = 63)		No intra-operative NMB monitoring (*n* = 177)		*P* value
	TOFR > 0.9	RNMB	TOFR > 0.9	RNMB	
Non-reversed (*n* = 165)	11/12	1/12	101/153	52/153	0.106#
Neostigmine (*n* = 14)	9/9	0/9	3/5	2/5	0.11#
Sugammadex (*n* = 61)	42/42	0/42	16/19	3/19	0.028#

*RNMB* Residual neuromuscular blockade

#Fisher’s exact test

Multivariable analysis revealed that the use of intra-operative quantitative NMB monitoring and the use of sugammadex were associated with a lower incidence of RNMB, with calculated ORs of 0.04 (95% CI: 0.005 to 0.401) and 0.18 (95% CI: 0.046 to 0.727), respectively. A longer period since last NMBD administration and the presence of a TOFR at the PACU was also associated with a lower incidence of RNMB (OR, 0.98; 95% CI: 0.977 to 0.995; Table 4).

**Table 4:** Multivariable logistic regression analysis for the association between residual neuromuscular blockade and potentially related factors

	OR	95% CI	*P* value
Intra-operative NMB monitoring	0.043	0.004 to 0.400	0.006
Sugammadex	0.182	0.045 to 0.727	0.016
Neostigmine	0.798	0.124 to 5.099	0.812
Duration of surgery	1.002	0.995 to 1.009	0.522
Time from last NMBD dose	0.986	0.977 to 0.995	0.002
Rocuronium	0.861	0.174 to 4.247	0.855
Atracurium	1.846	0.349 to 9.751	0.470

*OR* Odds ratio, *CI* Confidence interval, *NMB* Neuromuscular blockade, *NMBD* Neuromuscular blocking drug

## Discussion

The incidence of RNMB in patients without NMB monitoring in the present study (32%) is similar to the incidence reported in previous studies [15–17]. Moreover, the incidence of RNMB in monitored patients was significantly lower (1.6%; *P* <  0.01) than in non-monitored patients. These results are corroborated by substantial evidence suggesting that the use of intra-operative quantitative NMB monitoring is associated with a decreased incidence of RNMB in the PACU [18, 19].

The rate of quantitative NMB monitoring in the current study was 26%, which is very low considering the availability of monitoring equipment in every operating room. As Todd et al. [20] described, we believe the low NMB monitoring rates could be related to a poor understanding of the pharmacology of nondepolarising NMBDs. Additionally, very few adverse respiratory events in the PACU are confirmed to be a consequence of RNMB; hence, it is often not considered a clinically relevant problem and anaesthesiologists remain reluctant to use NMB monitoring.

Fifty-one of the 63 monitored patients (81%) received a reversal agent, while only 24 of the 177 nonmonitored patients (14%) received such an agent in our study. This shows that anaesthesiologists who do not use monitoring despite its availability probably do not consider the need for NMB reversal agents. On the contrary, anaesthesiologists who apply NMB monitoring routinely are more likely to consider peri-operative NMB management as an important aspect of patient safety and quality of care. These physicians likely administer reversal agents, whenever needed, following NMB monitoring results.

It is established that the spontaneous recovery of neuromuscular function (at least 4 responses measured by TOF) is crucial for successful antagonism with neostigmine [21]. As this is a retrospective study, we did not have access to data about the depth of paralysis at which neostigmine was administered. The incidence of RNMB in patients who received neostigmine in the nonmonitored group was 40 and 0% in the monitored group. A recent study showed a significant decrease in the incidence of severe postoperative RNMB in patients who received neostigmine in adjusted doses 10 min after a TOF count of 4 was confirmed at the thumb. However, RNMB was not completely prevented [22, 23]. Administration of neostigmine at an incorrect depth of paralysis could be a reason for failure to negate RNMB in the absence of NMB monitoring in the current study.

Our results show that sugammadex administration in the absence of NMB monitoring is not an effective strategy to avoid RNMB (16% incidence of RNMB). In these cases, reversal failure was likely due to an insufficient dosage, assuming the anaesthesiologists’ underestimation of the NMB depth. Although Kotake et al. [12] reported a lower incidence than noted in the present study for reversal failure with sugammadex in nonmonitored patients (4.3%; interquartile range: 1.7 to 9.4%), they also state that an incidence as high as 9.4% is not acceptable; therefore, sugammadex should not be used in the absence of NMB monitoring.

The current study had some limitations. First, it was an observational single-centre study conducted in a tertiary referral university hospital, rendering it difficult to generalise the applicability of the study results. Second, physicians in charge of TOFR measurements in the PACU were not blinded to the type of NMBD used, use of intra-operative NMB monitoring, or use of reversal agents. Third, 2 different monitors were used for intra-operative and postoperative periods because of limited availability. Fourth, the sample size for patients who received antagonists was not adequate to draw conclusions regarding this aspect. Last, we did not collect patient temperature data during the surgery or in the PACU, which may have affected the TOF measurements. Although the multivariable regression model showed an association between the use of NMB monitoring and the use of sugammadex, and a lower incidence of RNMB, the CIs of both ORs overlap. Consequently, we cannot state whether monitoring or sugammadex has a greater influence on the incidence of RNMB.

One patient in the monitored group exhibited RNMB. The patient presented in the PACU with an average TOFR (3 consecutive stimuli) of 0.87, and before extubation showed no apparent need for reversal. Despite having no electronic data available on TOFR results prior to extubation, it was verbally stated that all monitored patients who were extubated in the operating room had presented acceptable recovery of neuromuscular function, defined as TOFR > 0.9. Although TOFR results in the PACU should not be discordant because different monitors were used, the results could vary because of the patient’s wakefulness and responses to noxious stimuli.

All measurements were conducted when patients were conscious. Stimulation currents of ≤50 mA are acceptable in terms of patient comfort [24]. All TOFR measurements made in the PACU were obtained without prior calibration. There have been critical appraisals of the use of the TOF-Watch SX monitor without prior calibration. This monitor’s baseline configuration values provide both supramaximal stimulation and appropriate sensitivity for most typical adult patients. Also, this monitor has been used without calibration in several research studies [15, 24, 25]. We believe it is unlikely that calibration prior to our measurements would have modified the results of the present study.

Regarding the accuracy of TOFR measurements in conscious patients, it appears that 2 successive measurements may not be reliable; [26] however, some authors were able to report consecutive, stable, and concordant results using 3 TOFR measurements, similar to the protocol followed at our institution [13].

## Conclusions

The results of the present study suggest that intra-operative quantitative NMB monitoring and the use of sugammadex are associated with a decreased incidence of RNMB in the PACU, reinforcing the contention that the optimal strategy for RNMB avoidance is the use of quantitative NMB monitoring and the eventual use of reversal agents, if needed, prior to emergence from anaesthesia. Antagonism with either neostigmine or sugammadex without the use of NMB monitoring fails to prevent RNMB in the PACU. Further efforts should aim towards increasing awareness of RNMB and monitoring rates.

## Data Availability

The analysed data sets generated during the study are available from the corresponding author on reasonable request.

## Abbreviations

AMG: Acceleromyography
ASA: American Society of Anesthesiologists
CI: Confidence interval
NMB: Neuromuscular blockade
NMBD: Neuromuscular blocking drug
OR: Odds ratio
PACU: Postanaesthesia care unit
RNMB: Residual neuromuscular blockade
TOF: Train-of-four
TOFR: Train-of-four ratio
